# UNIFIED FRAMEWORK FOR MEASURING MOBILITY IN OLDER PEOPLE: EMERGING DATA FROM A CANADIAN AGING COHORT

**DOI:** 10.1093/geroni/igae098.0740

**Published:** 2024-12-31

**Authors:** Marla Beauchamp

**Affiliations:** McMaster University, Hamilton, Ontario, Canada

## Abstract

In the 2015 World Health Organization (WHO) World Report on Aging and Health, mobility is described as movement in all its forms either powered by the body or by a vehicle. Mobility thus encompasses basic movements such as standing up from a chair to more complex activities such as walking or using transportation. Many outcome measures have been developed to assess mobility, however, the variability in constructs being assessed and lack of standardisation in terminology present challenges to advance research and practice in this area. We propose a unified framework for mobility measurement in older populations consistent with the latest language and terminology endorsed by the WHO. The framework outlines three distinct constructs: perceived mobility (“what can you do?”), locomotor capacity for mobility (“what could you do?”), and actual mobility (“what do you do in daily life?”). The latter construct, actual mobility, has been less well studied until recently. Wearable devices offer the unique advantage of comprehensively monitoring the real-world actual mobility of older people in their homes and communities. In this talk, we will present emerging data from the McMaster Monitoring My Mobility (MacM3) study, a digital mobility cohort of over 1200 community-dwelling older adults with detailed mobility measurements spanning all three aspects of mobility and examine their relationships with clinically important health outcomes. In this way, we will be able to test the suitability of our framework and highlight the potential of digitally derived measures of actual mobility for informing aging research and practice.

